# Prolonged Nutrient Enrichment Slows the Recovery of Biodiversity and Productivity After Its Cessation

**DOI:** 10.1111/gcb.70981

**Published:** 2026-07-10

**Authors:** Miao He, Kathryn E. Barry, Elizabeth T. Borer, Yann Hautier, Cristy Portales‐Reyes, Eric W. Seabloom, David Tilman, Qianna Xu, Forest Isbell

**Affiliations:** ^1^ Department of Ecology, Evolution, and Behavior University of Minnesota St. Paul Minnesota USA; ^2^ Department of Biology, Institute of Science, Ecology and Biodiversity Utrecht University Utrecht the Netherlands; ^3^ Department of Biology Saint Louis University St. Louis Missouri USA; ^4^ Bren School of Environmental Science and Management University of California, Santa Barbara Santa Barbara California USA; ^5^ Washington State Department of Ecology Lacey Washington USA

**Keywords:** aboveground biomass, biodiversity‐ecosystem functioning, eutrophication, grassland restoration, long‐term recovery, nutrient cessation, nutrient legacy, nutrient policy

## Abstract

Many ecosystems worldwide are experiencing chronic anthropogenic nutrient enrichment, which often increases plant productivity while reducing species richness. Although nutrient inputs are now declining in some regions, the potential benefits of this reduction depend on the reversibility of enrichment impacts. In turn, ecosystem recovery can be determined by the enrichment history, that is, the rate and duration of nutrient enrichment. Here, we quantify how nutrient enrichment history shapes community recovery dynamics using a four‐decade grassland experiment that examines the joint effects of nutrient enrichment rate and duration with: (1) three durations of nutrient enrichment and recovery: one decade of enrichment followed by three decades of recovery, three decades of enrichment followed by one decade of recovery, or continuous enrichment for four decades; and (2) nutrient enrichment at a gradient of rates ranging from atmospheric deposition to agricultural fertilization. Our results showed nutrient enrichment increased plant biomass and reduced species richness, with higher nutrient addition rates leading to more rapid and sustained species loss and biomass increase, even over short enrichment periods. We assessed recovery dynamics following cessation as increases in species richness and declines in community biomass relative to control conditions, because of the tight coupling between richness and biomass in many communities. We found that the reversibility of enrichment effects depended on enrichment duration, with prolonged enrichment slowing recovery of both species richness and biomass, especially at high enrichment rates. However, biomass recovered more rapidly than species richness following cessation. These findings highlight that recovery trajectories of biodiversity and ecosystem functioning depend jointly on enrichment rate and duration, underscoring the need for restoration strategies that account for nutrient legacies and their determinants.

## Graphical abstract

Grassland recovery following nutrient cessation is asymmetric and strongly shaped by nutrient enrichment history. Higher nutrient addition rates and longer enrichment duration caused larger departures from control conditions, with biomass recovering more rapidly than species richness over decadal timescales.

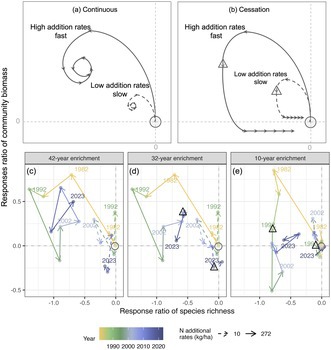

## 摘要

全球范围内许多生态系统正在经历由长期人类活动引起的营养物质富集，这往往会提高植物群落生产力，同时降低物种丰富度。尽管部分地区的营养物质输入正在减少，但这种减少能否带来生态效益，取决于营养物质富集效应的可逆性。而生态系统恢复的过程又可能受到营养物质富集历史的影响，即营养物质添加的速率与持续时间。本研究基于一项持续四十余年的草地实验，量化了不同营养物质富集历史如何影响植物群落恢复动态。该实验同时控制了养分添加速率与持续时间，包括:(1)三种不同的养分添加与恢复历史:添加10年后停止并恢复32年、添加32年后停止并恢复10年，以及持续添加42年；(2)八种养分添加速率，覆盖从大气氮沉降到农业施肥水平(0–272 kg N·ha⁻¹·yr⁻¹)。 研究结果表明，即使在较短的富集时间内，较高的养分添加速率也会导致更快速且持续的物种丧失与生物量增加。由于许多植物群落中物种丰富度与生物量之间存在紧密耦合关系，养分添加停止后，我们通过物种丰富度以及群落生物量相对于对照条件的差距来衡量群落恢复动态。研究发现，营养物质富集效应的可逆性受到养分添加持续时间的显著影响；长期养分添加会延迟物种丰富度与群落生物量的恢复，尤其是在高养分添加速率下更为明显。不过，在停止养分添加后，群落生物量的恢复速度显著快于物种丰富度。这些结果表明，生物多样性与生态系统功能的恢复轨迹受到养分添加速率与持续时间的共同影响，凸显了充分考虑营养物质遗留效应及其决定因素对于生态恢复策略制定的重要性。

## 1 Introduction

Nitrogen deposition and fertilization have caused widespread eutrophication across terrestrial ecosystems, with substantial variation in enrichment rates and duration across regions (Galloway et al. 2003; Penuelas et al. 2020; Peñuelas and Sardans 2022; Stevens et al. 2010). Although elevated nutrient availability can increase primary production (Harpole et al. 2011; Lawes and Gilbert 1880; Tilman 1987b; Vitousek and Howarth 1991), nutrient enrichment also tends to reduce plant diversity (Harpole et al. 2016; Seabloom, Adler, et al. 2021; Seabloom, Borer, et al. 2021; Stevens et al. 2004; van der Plas et al. 2024). Importantly, these impacts can accumulate over decades through persistent changes in soil properties (Johnson et al. 2003; McLauchlan 2006; Seabloom et al. 2025), species pools (Eskelinen et al. 2023; Wacker et al. 2009; Yang et al. 2024), and competitive hierarchies (Dybzinski and Tilman 2007; Hautier et al. 2009; Peng et al. 2025; Tilman 1982), often persisting long after nutrient inputs decline. While policy changes have reduced nutrient inputs in some regions, particularly atmospheric nitrogen deposition (Berendse et al. 2021; Borer and Stevens 2022; Schoukens 2017), the effectiveness of nutrient reduction depends on the reversibility of enrichment impacts (Clark and Tilman 2008; Isbell, Reich, et al. 2013; Isbell, Tilman, et al. 2013; Storkey et al. 2015), which may be determined by both the rate and the duration of nutrient enrichment (Berendse et al. 2021; Niu et al. 2022; Seabloom, Adler, et al. 2021; Seabloom, Borer, et al. 2021). However, how nutrient addition rate and duration jointly shape long‐term recovery dynamics remains unclear. The lack of knowledge of these effects limits our ability to predict long‐term biodiversity recovery and guide ecosystem management under global changes.

The duration of nutrient enrichment can determine the magnitude of impacts (Niu et al. 2022; Seabloom, Adler, et al. 2021; Seabloom, Borer, et al. 2021; Yang et al. 2021). Short‐term enrichment may cause transient increases in biomass or shifts in dominance (Song et al. 2011, 2019), whereas long‐term enrichment can alter soil conditions, increase dominance by ruderal species, and drive species losses (Bever et al. 1997; Isbell, Reich, et al. 2013; Seabloom, Adler, et al. 2021; Seabloom, Borer, et al. 2021; Smith et al. 2009). The rate of enrichment can also determine the magnitude of impacts: high enrichment rates can rapidly increase biomass and dominance by fast‐growing species and trigger species loss through competitive exclusion, whereas low enrichment rates may produce weaker or delayed effects (Isbell, Tilman, et al. 2013; Namuhan et al. 2024; Song et al. 2011; Tilman 1987a). Although rate and duration are often studied separately, their joint effects on both the magnitude and reversibility of enrichment impacts remain poorly understood (Carpenter et al. 1999; Mountford et al. 1996; Seabloom et al. 2020; Tilman and Isbell 2015; Yang et al. 2024).

The impacts of nutrient enrichment on grassland biomass and species richness might also be coupled (Duprè et al. 2010; Isbell, Reich, et al. 2013; Namuhan et al. 2024; Seabloom, Adler, et al. 2021; Seabloom, Borer, et al. 2021; Weigelt et al. 2009). A nutrient‐induced increase in biomass can lead to a consequent decrease in richness through intensified light competition (Borer et al. 2014; DeMalach et al. 2017; Dybzinski and Tilman 2007; Hautier et al. 2009; Namuhan et al. 2024), often shifting communities from a diverse and productive native assemblage toward domination by a few nutrient‐limited non‐native species (Chapin et al. 1986; Harpole and Tilman 2007; Kaul and Wilsey 2021; Seabloom, Adler, et al. 2021; Seabloom, Borer, et al. 2021). Conversely, a decrease in species richness can reduce biomass through reduced niche complementarity, though such effects are context‐dependent and may emerge only over longer timescales (Kahmen et al. 2006; Loreau and Hector 2001; Mason et al. 2020; Roscher et al. 2016). Following cessation of nutrient enrichment, recovery trajectories of biomass and species richness might therefore exhibit asymmetric dynamics: slow recolonization of lost species (Milchunas and Lauenroth 1995; Pichon et al. 2023; Reyes‐Portales 2020; Schmid 2002; Yang et al. 2024) or persistent soil nutrient legacies (O'Sullivan et al. 2011; Seabloom et al. 2025) may delay richness recovery, which in turn can influence how it covaries with the biomass recovery over decadal timescales (Eskelinen et al. 2022; Hautier et al. 2018; Isbell et al. 2019). Despite the relevance of species richness and community biomass for ecosystem recovery and management, it remains unclear whether these responses recover to no‐enrichment conditions at similar rates following prolonged enrichment, and how their recovery depends on enrichment history.

To address these gaps, we used a four‐decade experiment at the Cedar Creek Ecosystem Science Reserve in Minnesota, USA (Tilman 1987b; Wedin and Tilman 1996). This experiment created a gradient of nutrient addition rates (0–272 kg N/ha/year in combination with other nutrients) crossed with three nutrient enrichment durations: (1) cessation after 10 years (1982–1991) of enrichment, (2) cessation after 32 years (1982–2013) of enrichment, and (3) continuous enrichment for 42 years (1982–2023). Earlier studies based on this experiment found that after one decade of low to moderate nutrient enrichment (0–95 kg N/ha/year), plant diversity recovered within one decade following cessation (Clark and Tilman 2008); whereas after one decade of high‐rate nutrient enrichment (95–272 kg N/ha/year), plant diversity remained low even after two decades of recovery (Isbell, Tilman, et al. 2013). This study adds an additional decade of this experiment and a more recent cessation treatment, which allows us to address two questions: (1) how do nutrient enrichment rate and duration jointly determine the magnitude and reversibility of impacts on plant species richness and community biomass? (2) do richness and biomass recover at similar rates following cessation, or does enrichment history generate asymmetric recovery dynamics?

## 2 Methods

### 2.1 Experimental Design

In a four‐decade grassland experiment at Cedar Creek Ecosystem Science Reserve in Minnesota, USA, we applied long‐term nutrient enrichment with varying nutrient addition rates and cessation treatments since 1982 (Tilman 1987b; Wedin and Tilman 1996). The experimental site was established in a successional grassland abandoned from agriculture in 1934, with all plots tilled at the start of the experiment in 1982 (Tilman 1987b). The site has infertile, sandy soils and a mean annual precipitation of 682 mm, a mean annual temperature of 7.5°C (WeatherSpark 2025), and a background atmospheric N deposition of approximately 9 kg N/ha/year (Ackerman et al. 2019). In this experiment, there is one treatment with no nutrient addition (control) and eight treatments with the addition of non‐nitrogen (N) nutrients in combination with N (in the form of pelletized NH_4_NO_3_) at eight different rates: 0, 10, 20, 34, 54, 95, 170, or 272 kg N/ha/year (Clark and Tilman 2008), which simulates N enrichment from a continuum from atmospheric deposition to agricultural fertilization (Ackerman et al. 2019; Adalibieke et al. 2023). All fertilized plots received P, K, Ca, Mg, and trace metals in addition to N to ensure the system was primarily N‐limited (Tilman 1987b). These additional nutrients were discontinued together with N in cessation treatments. Unamended control plots received no nutrients and were distinct from plots that received zero N but other nutrients. Additional details of the experimental design can be found in Tilman (1987b), Seabloom, Adler, et al. (2021), and Seabloom, Borer, et al. (2021).

The study has a total of 54 plots (4 m by 4 m) with 1 m buffers between plots [(eight nutrient addition rates + one unamended control) × six replicates] (see Figure S1 for spatial layout). In 1992, three of the six replicates per nutrient enrichment treatment, which had received 10 years of nutrient enrichment, were randomly assigned to stop fertilization. In 2014, three remaining replicates, which had received 32 years of nutrient enrichment, were split into two subplots: a fertilized subplot (4 m × 1.5 m) and a cessation subplot (4 m × 2.5 m). The side (west or east) of subplots assigned to continued fertilization was determined randomly by coin flip.

We monitored plant species richness and species‐specific peak aboveground biomass in all plots by clipping with 10 cm by 300 cm strips placed at least 50 cm from the plot edge (Tilman 2025). The experimental area (Field C) is fenced and unburned, with no manual biomass removal other than the sample collection within the strips. Biomass was collected and dried annually from 1982 to 1994, in at least two of every 3 years from 1995–2004 and 2015–2023, as well as in 2008 and 2011. We recorded a total of 142 species (including 11 unidentified species), with each sampling strip containing 1 to 24 species in a given year. Woody species accumulate aboveground biomass across years and are therefore not directly comparable to the annual aboveground production of herbaceous biomass, which resets following snowmelt each growing season at this temperate site. Additionally, woody species represent a relatively small proportion of all recorded species (10.94%) and did not show systematic responses to nutrient treatments or through time. Thus, woody–herbaceous interactions are unlikely to drive the nutrient‐related patterns in species richness and community biomass observed here. Therefore, 14 woody species were excluded from the analysis.

### 2.2 Statistical Analysis

All statistical analyses were conducted in R (version 4.2.0). To examine the temporal trends in plant species richness and community biomass at the plot level, we calculated the number of plant species and total aboveground biomass for each plot in each sampling year. Temporal trends were visualized by fitting loess regressions of community biomass against the calendar year using the geom_smooth function in the *ggplot2* package (Figure S2; Wickham 2016). To test a priori hypotheses about responses of species richness and community biomass to nutrient addition, we used linear mixed‐effects models that account for continuous gradients in nutrient addition rate and repeated measurements within plots. Nutrient addition rate was treated as a log‐transformed continuous variable (Nlevel), whereas nutrient enrichment duration (cessation after 10 or 32 years of enrichment, or continuous enrichment for 42 years) was treated as a categorical variable (Nduration) in all analyses.

**FIGURE 1:**
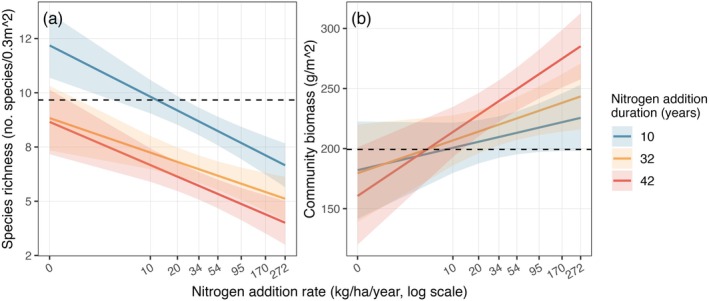
Species richness (a) and community biomass (b) changes with increasing nitrogen addition rates (+PK) under different nutrient addition durations. Plots receiving continuous fertilization were fertilized for 42 years (red), whereas plots under cessation treatments received 10 (blue) or 32 years (orange) of nutrient addition before cessation. Lines represent model‐predicted absolute values from linear mixed‐effects models with log‐transformed nutrient addition rate, and shaded areas indicate 95% confidence intervals. Dashed horizontal lines indicate the mean values of unamended control plots (no nutrient addition).

To compare current species richness and community biomass among nutrient histories, we analyzed data from the three most recent sampling years (2019, 2022, 2023), during which both variables showed no consistent temporal trends (Figure 1). We fitted linear mixed‐effects models separately for species richness and community biomass using the *lme4* package (Bates et al. 2014). Models included log‐transformed nutrient addition rate, duration, and their interaction as fixed effects, and plot as a random effect to account for repeated measurements:

(Model 1) $$\;\text{lmer}\left(y\sim \log{\left(\text{Nlevel}+1\right)}^{*}\;\text{factor}\left(\text{Nduration}\right)+\left(1\;|\;\text{Plot}\right)\right)$$

To assess potential deviation from log‐linear responses, we additionally fitted models including both continuous (log‐transformed) and categorical terms for nutrient addition rate and duration:

(Model 2) $$\begin{array}{c} \text{lmer}\left(y\sim {\left(\log\left(\text{Nlevel}+1\right)+\text{factor}\left(\text{Nlevel}\right)\right)}^{*}\right.\\ \left.\;\left(\text{Nduration}+\text{factor}\left(\text{Nduration}\right)\right)+\left(1\;|\;\text{Plot}\right)\right) \end{array}$$

Because categorical terms did not provide strong evidence for deviations from continuous responses, we focused on Model 1 for inference. Type I analysis of variance (ANOVA) was used to test the effects of nutrient addition rate, duration, and their interaction based on Model 1 (Table S1) and Model 2 (Table S2). We also conducted post hoc comparisons with unamended control plots using Dunnett‐adjusted tests (Dunnett 1955, 1964), and estimated mean values for each treatment were calculated using the *emmeans* package (Lenth et al. 2022; Figure S3).

To quantify recovery following cessation of nutrient addition, we evaluated the recovery of species richness and community biomass toward levels observed in unamended control plots, as a function of prior nutrient addition rate and duration (Figure S5). Linear mixed‐effects models were fitted separately for species richness and community biomass, with log‐transformed nutrient addition rate, duration, and their interaction as fixed effects. Year was included as a fixed effect to test for directional temporal trends and as a random factor to account for shared interannual variation among plots. Plot was included as a random factor to account for repeated measurements:

(Model 3) $$\begin{array}{c} \text{lmer}\left(y\sim \log{\left(\text{Nlevel}+1\right)}^{*}\;\text{factor}\left(\text{Nduration}\right)+\text{Year}\right.\\ \;\left.+\left(1\;|\;\text{Plot}\right)+\left(1\;|\;\text{Year}\right)\right) \end{array}$$

Model assumptions of normality and homoscedasticity were assessed using residual diagnostics. Type I ANOVA was used to test the significance of nutrient addition rate, duration, and their interaction based on Model 3 (Table S3).

**FIGURE 2:**
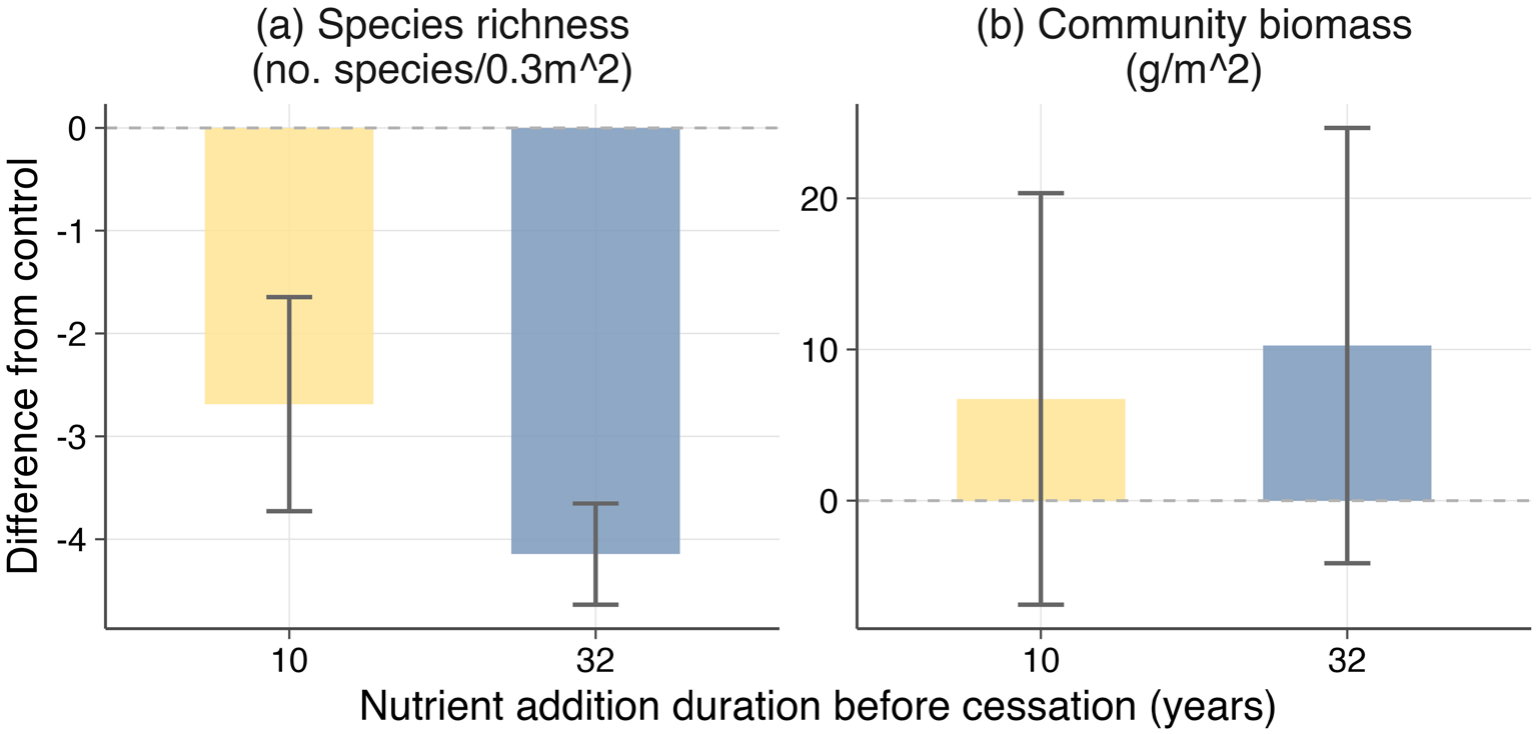
Differences in species richness (a) and community biomass (b) from unamended control conditions in the first decade following 10 (yellow) or 32 (blue) years of nutrient enrichment. Bars show the differences between model‐estimated means (with standard errors) and the unamended controls (no nutrient addition) across all nutrient addition rates. Dashed horizontal lines indicate the unamended control conditions; closer to the line indicates more recovery towards the non‐addition level. Values are centered at zero to represent deviation from unamended control conditions, absolute values can be found in Figure S2.

To further compare recovery following different durations of nutrient addition, we evaluated the relative and absolute recovery of species richness and community biomass changes one decade following cessation of 10‐year and 32‐year addition (Figures 2, S6 and S7). Relative recovery was quantified as the difference between cessation plots and unamended control plots (cessation plot—control plot) at a comparable time point one decade after each cessation (Figure 2), accounting for a shifting baseline. Absolute recovery was quantified as the changes within plots over the first decade following each cessation (later year—former year) for both cessation and control plots (Figures S6 and S7), evaluating the degree of returning to the past conditions. Instead of using time since cessation as a predictor, these comparisons use a fixed time window (one decade, the maximum time following the later cessation) to quantify how recovery depends on nutrient addition duration, which allows us to control for interannual variation.

To examine how the relationship between species richness and community biomass changes over time, we quantified temporal variation in biomass–richness relationships across control, fertilized, and recovery phases (Figure S8). The fertilized phase includes plots receiving continuous nutrient enrichment as well as the period before cessation in plots that stopped nutrient enrichment, and the recovery phase includes the period following nutrient cessation in those plots. For each year (and for each cessation treatment in the recovery phase), we fitted linear models of community biomass as a function of species richness and extracted the slope as a measure of the biomass–richness relationship.

**FIGURE 3:**
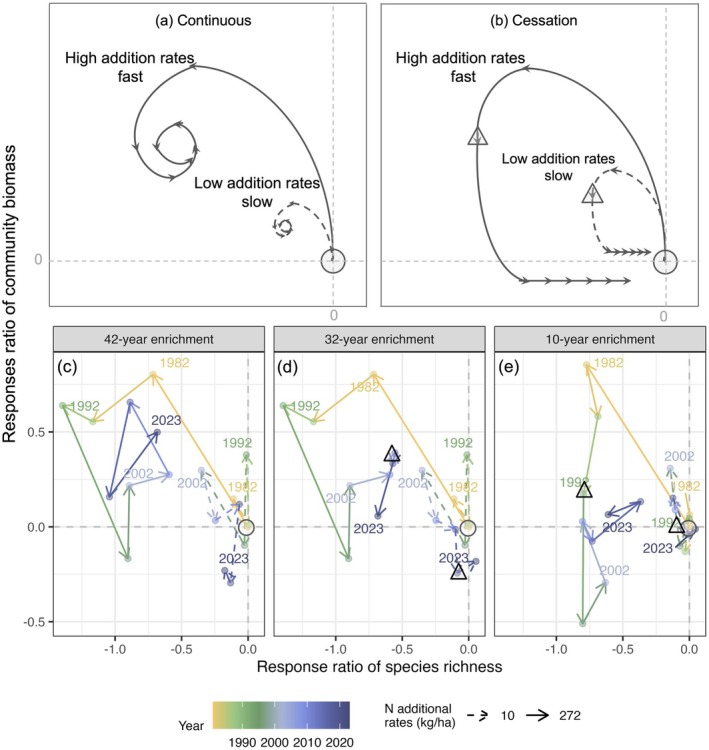
Hypothesized (a, b) and observed (c–e) temporal trajectories of species richness and community biomass under continuous nutrient enrichment (a, c) and following nutrient cessation (b, d, e). Trajectories are shown in a richness–biomass response‐ratio space relative to the unamended control (open circles at the origin; no nutrients were added in the unamended control plots, distinct from the plots with nutrient addition rate “0” where no N but other nutrients were added). Dashed horizontal and vertical gray lines indicate the unamended control conditions of community biomass and species richness, respectively. Triangles represent the years of cessation, where applicable (d, e). Panels (c–e) show trajectories for representative low (10 kg N/ha/year; dashed lines) and high (272 kg N/ha/year; solid lines) nutrient addition rates. Results of all rates are shown in Figure S9. Arrows represent equal time intervals, with color indicating the sampling year.

To further examine the responses of species richness and community biomass to nutrient enrichment independent of absolute magnitudes and interannual variability, we calculated their responses relative to control plots across the full period of available data (1982–2023) (Figures 3, S4 and S9). Log response ratios (LRR) were calculated as $\mathrm{LRR}=\log\left(Y_{t}/Y_{c}\right)$, where $Y_{t}$ and $Y_{c}$ represent the mean values in enriched and control plots, respectively. The sampling variance of each LRR (LRR_var) was estimated from the variance, mean, and sample size of the treatment and control groups using the *metafor* package (Viechtbauer 2010). Linear mixed‐effects models were fitted separately for LRR (species richness) and LRR (community biomass), with log‐transformed nutrient addition rate, duration, and their interaction as fixed effects. Year was included as a fixed effect to test for directional temporal trends and as a random intercept to account for year‐specific deviations shared across observations within the same year. Sampling variances were incorporated through a fixed variance structure, and the residual variance was fixed to 1 to reflect known observation‐level uncertainty. Models were fitted using the lme function in the *nlme* package (Pinheiro et al. 2025; Pinheiro and Bates 2000):

(Model 4) $$\begin{array}{c} \mathrm{lme}\left(\mathrm{LRR}\sim \log{\left(\text{Nlevel}+1\right)}^{*}\;\text{factor}\left(\text{Nduration}\right)\right.\\ \left.+\text{Year},\text{random}=\sim 1|\text{Year},\text{weights}\right.\\ \left.=\text{varFixed}\left(\sim \mathrm{LRR}\_\mathrm{var}\right),\text{control}=\text{lmeControl}\left(\text{sigma}=1\right)\right) \end{array}$$

When visualizing temporal trajectories of richness–biomass responses (Figures 3 and S9), LRRs were averaged within a 5‐year window to reduce the influence of interannual variability and uneven sampling frequency; annual responses were presented in Figure S4. Type I ANOVA was used to test the significance of nutrient addition rate, duration, and their interaction based on Model 4 (Table S4).

To assess whether ecosystem responses were driven by a few extreme values (a small number of plots dominated by *Urtica dioica* ), we conducted sensitivity analyses excluding plots that exhibited consistently high dominance (> 80%) of this species across multiple years. Excluding these plots did not change the direction or significance of temporal trends in species richness and community biomass, nor did it alter community responses to nutrient enrichment.

## 3 Results

Nutrient addition reduced species richness and increased community biomass, and the strength of these effects increased with nutrient addition rate (Figures 1, S2 and S3). The effect of nutrients on species richness increased with the nutrient addition duration (i.e., the number of years the treatment was applied), but the effect of nutrient addition rate remained constant over time, providing little evidence for an interaction between nutrient addition rate and duration (Figure 1a; Table S1). In contrast, the effect of nutrient addition rate on biomass increases with duration, suggesting some evidence of rate‐duration interaction (Figure 1b; *p* < 0.1, Table S1). Nutrient addition reduced species richness rapidly once applied, particularly at higher enrichment rates, and the effect remained with prolonged fertilization. In contrast, nutrient addition increased biomass initially, but this increase diminished over time and biomass even declined in recent years (Figures S2 and S3). However, there was no significant effect of year, indicating that neither species richness nor community biomass exhibited a consistent temporal trend under nutrient enrichment (Figure S5; Table S3).

Higher rates of nutrient enrichment slowed the recovery after cessation (Figures S2 and S3). Following a decade of high‐rate nutrient enrichment (95–272 kg N/ha/year), species richness did not begin to recover until the third decade after enrichment stopped, and richness still had not returned to control conditions by the fourth decade (Figures S2b and S3a). In contrast, after one decade of nutrient enrichment at moderate rates (0–54 kg N/ha/year), species richness recovered to control conditions within the first decade following cessation and remained higher thereafter (Figure S3a). However, after three decades of nutrient enrichment at the same moderate rates, species richness failed to recover during the first decade following cessation (Figures S2c and S3a). Community biomass converged more rapidly toward the unamended control level than species richness following cessation, particularly after longer enrichment duration (Figures S2f,g and S3b).

Relative recovery (i.e., difference from unamended control levels measured in the same year) indicated strong legacy effects of enrichment duration on species richness and community biomass (Figure 2). One decade following cessation, species richness and biomass in communities that had received nutrients for one decade were substantially closer to unamended control conditions than communities that had received nutrients for three decades (Figure 2). When recovery was quantified as absolute change since the cessation year (later year value minus cessation‐year value), species richness exhibited only modest net change with substantial uncertainty over the first decade following cessation after both nutrient addition durations (Figure S6a). Recovery of species richness showed weak and inconsistent changes across nutrient addition rates, indicating limited short‐term recovery along the nutrient gradient (Figure S7a). In contrast, community biomass declined more strongly at higher nutrient addition rates following cessation, especially after long‐term enrichment (Figure S7b). Biomass in the 10‐year enrichment plots returned to approximate control conditions within a few years, whereas the 32‐year enrichment plots still supported slightly higher biomass than controls after one decade of recovery, despite large declines from their fertilized peak (Figure S4).

Consistent with absolute‐scale analysis (Figures S4 and S8), species richness declined and community biomass increased with increasing nutrient addition rates and duration when expressed as log response ratios (LRR), indicating that these patterns were robust to differences in absolute magnitude and interannual variability (Figures 3 and S4). However, LRR (species richness) showed no significant temporal trend (Table S4), indicating that the richness responses were largely independent of temporal dynamics and primarily structured by ongoing or prior nutrient enrichment. In contrast, LRR (community biomass) declined significantly over time, with a significant interaction between nutrient addition rate and duration (Table S4), which indicates that accounting for baseline variation reveals time‐dependent biomass responses that are not evident in absolute‐scale analyses (Figure S8). These asymmetric patterns were further reflected in the temporal trajectories of species richness and community biomass in response‐ratio space: LRR (species richness) and LRR (community biomass) followed distinct and nutrient‐history‐dependent recovery trajectories after nutrient enrichment ceased (Figures 3 and S9). Consistent with our hypotheses (Figure 3a,b), communities under continuous nutrient enrichment remained displaced from control conditions, with elevated biomass and reduced species richness (Figure 3c), and higher nutrient addition rates produced larger displacements and slower return of community responses to control conditions (Figure S9). Following cessation, LRR (community biomass) declined rapidly toward the control condition, whereas recovery of LRR (species richness) lagged behind (Figure 3d,e). The asymmetry of response trajectories further diverged with enrichment duration: in plots fertilized for one decade before cessation, species richness showed a slow but directional increase toward the unamended control after biomass declined (Figure 3d); whereas in plots fertilized for three decades, species richness showed little evidence of recovery over the same period despite substantial biomass declines (Figure 3e).

## 4 Discussion

Our study reveals both rate‐ and duration‐dependent legacy effects on the recovery of community diversity and ecosystem functioning following nutrient cessation. Prolonged nutrient enrichment slowed the recovery of both species richness and community biomass after nutrient enrichment ended, and higher enrichment rates further exacerbated these duration effects. Species richness and community biomass followed distinct and asymmetric recovery trajectories, with longer duration and higher rate prior to cessation leading to greater divergence from control conditions as well as slower recovery toward control conditions. Together, these results show that nutrient legacies depend not only on how much nutrient is added but also on how long enrichment persists, and that biodiversity and ecosystem function recover on decoupled temporal scales.

Both the magnitude of biodiversity loss and production enhancement under nutrient enrichment as well as their rates of recovery depended on nutrient addition rate and duration, with higher and longer inputs leading to stronger declines and slower recovery following cessation (Figure 1; Table S1). Species richness declined gradually in unamended control plots (Figure S2a), which could result from background atmospheric nitrogen deposition of approximately 10 kg N/ha/year (Ackerman et al. 2019), ongoing succession after historical tilling (DeSiervo et al. 2023; Tilman 1987b), or the absence of mowing or burning (Borer et al. 2020; Reyes‐Portales 2020). At low enrichment rates (including 0 N + PK), species richness remained comparable to control conditions throughout four decades of this experiment (Figure S4), likely reflecting the combined influence of background atmospheric nitrogen deposition and additional non‐N nutrient inputs. While lower enrichment rates caused weaker declines in species richness and allowed its gradual recovery following cessation (Clark and Tilman 2008; Isbell, Tilman, et al. 2013), higher nutrient addition rates led to more rapid and sustained species loss (Figures 1a and S2). High rates of nutrient addition also led to larger increase in community biomass, particularly during early enrichment stages, but this positive effect weakened under prolonged enrichment (Figures 1b and S2), consistent with diminishing returns in productivity under long‐term nutrient inputs (Isbell, Reich, et al. 2013; Kimmel et al. 2020). In contrast, prolonged nutrient enrichment led to larger species richness decline throughout the experiment period (Figure 1a), consistent with previous findings showing strong and sustained biodiversity loss (Clark et al. 2009; Hao et al. 2018; Seabloom, Adler, et al. 2021; Seabloom, Borer, et al. 2021). This sustained decline in species richness is likely reinforced by persistent soil legacies, including elevated soil C and N (Seabloom et al. 2025), altered soil chemistry and pH (Berendse et al. 2021; Fang et al. 2012; Kimmel et al. 2020; Niu et al. 2022), and soil degradation (Hu et al. 2024; Stevens et al. 2012; Tian and Niu 2015).

While nutrient addition rate largely determined the magnitude and short‐term trajectory of species richness and biomass responses, enrichment duration constrained recovery following cessation. Given the same recovery window of one decade, richness and biomass in plots that received longer enrichment prior to cessation remained substantially farther from unamended control conditions (Figure 2). This indicates that prolonged enrichment may slow community recovery through extended species loss, erosion of local species pools, and reduction in the availability of colonists after cessation (Gross et al. 2005; Hao et al. 2018; Lonardi et al. 2024; Niu et al. 2022), besides the sustained changes in soil properties discussed earlier. In contrast, a shorter duration of enrichment likely enabled a greater recovery potential, as many species may have persisted at low abundance or in the seed bank during enrichment and then rapidly proliferated following cessation, restoring diversity (Bai et al. 2025; Storkey et al. 2015). In long‐term enriched plots, a small number of nutrient‐sensitive species maintained high biomass after cessation, thereby continuing to suppress colonization by other species and maintaining a low‐diversity, high‐biomass state (Clark and Tilman 2010; Isbell, Tilman, et al. 2013; Zhang et al. 2023). Therefore, the duration of nutrient enrichment can determine the recovery rate of species richness and community biomass.

The divergence between relative recovery and absolute change revealed different aspects of post‐cessation dynamics. Relative recovery captures the extent to which communities remained different from reference conditions (i.e., unamended controls), as the reference conditions are shifting over time due to recovery from tilling (Figure S2a; DeSiervo et al. 2023; Tilman 1987b), which is relevant for restoration goals aimed at returning an ecosystem toward a target state. Through the lens of relative recovery, the 32‐year enrichment legacy left communities persistently farther from control richness than the 10‐year legacy after a decade (Figure 2), consistent with durable nutrient‐driven losses of diversity and slow reassembly of the species pool (Isbell, Reich, et al. 2013; Mason et al. 2011; Muehleisen et al. 2023). In contrast, absolute change, which reflects within‐community temporal trajectories following cessation and is sensitive to the initial conditions, may be small even when communities remain far from reference states (e.g., species richness of a community can remain far below control conditions with little change over a decade). In our experiment, the large relative deficits and modest absolute changes in richness during the first decade (Figures S6 and S7) suggest that passive recovery proceeds slowly once enrichment has reduced species richness and reinforced dominance by a few highly productive species, especially where long‐term fertilization has reduced seed bank or species pool, entrenched competitive dominance, or sustained altered soil conditions (Fang et al. 2012; Niu et al. 2022; Seabloom et al. 2025; Stevens et al. 2012). Together, these results imply that enrichment duration primarily constrains the state of recovery (distance from reference) rather than the rate of short‐term change over the first decade following cessation.

Nutrient addition rate and duration jointly lead to asynchronous recovery of species richness and community biomass (Figures 3 and S9). Trajectories under higher nutrient addition rates occupied larger regions of the richness‐biomass response space and progressed more slowly toward unamended control following cessation (Figure 3d,e), indicating stronger inertia and legacies under high‐rate enrichment (Hao et al. 2018; Seabloom et al. 2020; van Paassen et al. 2020). Changes in community biomass generally preceded detectable changes in species richness following cessation (Figure 3d,e). Such lags can be attributed to the loss of rare species under enrichment (Allan et al. 2025; Andraczek et al. 2024; Soliveres et al. 2016; Vogel et al. 2019), which often require decades to re‐establish due to dispersal and seed limitation (Eisenhauer et al. 2019; Pichon et al. 2023). This asymmetry and its dependence on nutrient addition history correspond to the temporal dynamics of biomass–richness relationships (Figure S8): while these relationships remained relatively stable in control plots over time (Figure S8a), they became less negative with increasing years of nutrient addition (Figure S8b), indicating increasing decoupling between biomass and species richness, with less peak biomass and greater species loss. Following cessation, slopes partially recovered toward more neutral relationships, with trajectories differing by enrichment duration (10 vs. 30 years of enrichment; Figure S8c). The slower recovery of species richness may not immediately translate into biomass gain, because of the positive richness‐productivity relationships observed in many grasslands, possibly due to reduced niche complementarity (Band et al. 2022; Kahmen et al. 2006; Roscher et al. 2016), altered nutrient resorption strategies (Lü et al. 2021), or slower litter decomposition in nutrient‐enriched soil (Clark and Tilman 2010; Gill et al. 2022; Reyes‐Portales 2020). In addition, our study field is neither mowed nor burned, and there is no biomass removal practice other than sample collection in the 10 cm x 300 cm strips. The accumulation of live and dead biomass under enrichment can inhibit seedling establishment through light limitation and litter drowning (Facelli and Pickett 1991; Fang et al. 2012; Hautier et al. 2009, 2018; Kortessis et al. 2022; Reyes‐Portales 2020), potentially delaying richness recovery even as biomass declines following cessation. These conditions may differ from more frequently disturbed grasslands, such as those in Europe, where biomass removal can facilitate biodiversity recovery (Storkey et al. 2015; Liu et al. 2025).

Our study demonstrates that the recovery of biodiversity and productivity following nutrient cessation is constrained by both the rate and duration of prior enrichment. Extended nutrient enrichment leaves persistent community and soil legacies that constrain recovery even decades after cessation (Isbell, Tilman, et al. 2013; Shi et al. 2014; Yang et al. 2024). These findings underscore the urgency of policies and practices to reduce both the intensity and duration of nutrient pollution (Liu et al. 2025; Manning et al. 2006; Resch et al. 2021), as delayed cessation not only prolongs recovery but also increases the need for costly restoration interventions (Mountford et al. 1996; Shipley et al. 2024). Additionally, the duration of nutrient enrichment can be a critical determinant of recovery trajectories; simply eliminating nutrient inputs may be insufficient to restore biodiversity in long‐eutrophied systems in the short term (Krause et al. 2016; Niu et al. 2022; Seabloom, Adler, et al. 2021; Seabloom, Borer, et al. 2021). Longer recovery periods or active restoration interventions (such as species reintroductions or soil amendments) may be necessary to overcome the entrenched low‐diversity state and achieve full recovery of plant species richness after prolonged nutrient enrichment (Isbell, Tilman, et al. 2013; Ladouceur et al. 2023; Resch et al. 2021; Schmid 2002; Strengbom et al. 2001). By disentangling nutrient addition rate and duration and linking these to long‐term recovery dynamics, our study highlights the importance of early intervention and duration‐aware management strategies for promoting the resilience and restoration of grassland ecosystems (Drever et al. 2021; Ellis et al. 2024; Krause et al. 2016; Munroe et al. 2013; Plantinga 1996).

## Author Contributions

**Kathryn E. Barry:** writing – review and editing, methodology. **Elizabeth T. Borer:** writing – review and editing, supervision. **Yann Hautier:** writing – review and editing, formal analysis, methodology. **Miao He:** conceptualization, methodology, formal analysis, writing – original draft, writing – review and editing, visualization. **Forest Isbell:** conceptualization, methodology, investigation, supervision, writing – review and editing. **Cristy Portales‐Reyes:** investigation, writing – review and editing. **Eric W. Seabloom:** writing – review and editing, methodology, formal analysis, supervision. **David Tilman:** methodology, investigation, funding acquisition, project administration, writing – review and editing. **Qianna Xu:** methodology, writing – review and editing.

## Funding

This work was supported by the National Science Foundation, DEB‐2425352, DEB‐1831944.

## Conflicts of Interest

The authors declare no conflicts of interest.

## Supporting information


**Figure S1:** Spatial layout of experimental plots (Field C in experiment 002 at Cedar Creek).
**Figure S2:** Temporal dynamics of plant species richness (a–d) and community biomass (e–h) under different nutrient addition rates and cessation treatments.
**Figure S3:** Estimated species richness (a) and community biomass (b) averaged across the three most recent sampling years (2019, 2022, 2023).
**Figure S4:** Temporal trajectories of log response ratios (RR) for species richness (red) and community biomass (blue) under different nutrient addition rates (from top to bottom panels) and cessation treatments (from left to right panels). Values are shown relative to unamended control plots.
**Figure S5:** Species richness (a, b) and community biomass (c, d) across nutrient addition rates after cessation of 10‐year enrichment (a, c) or 32‐year enrichment (b, d).
**Figure S6:** Changes in species richness (a) and community biomass (b) in the first decade following 10 (yellow) or 32 (blue) years of nutrient enrichment, as well as the corresponding changes in unamended control over the same periods (10_Control and 32_Control).
**Figure S7:** Changes in species richness (a) and community biomass (b) in the first decade following 10 (yellow) or 32 (blue) years of nutrient enrichment at different rates.
**Figure S8:** Temporal changes in the relationship between species richness and community biomass under control (a), fertilized (b), and recovery following nutrient cessation (c).
**Figure S9:** Temporal trajectories of species richness and community biomass responses under continuous nutrient enrichment and following nutrient cessation across all nutrient addition rates.
**Table S1:** ANOVA table of linear mixed‐effects models (Model 1) testing the effects of log‐transformed nitrogen addition rate (Nlevel), duration (Nduration), and their interaction on current species richness and community biomass.
**Table S2:** ANOVA table of linear mixed‐effects models (Model 2) testing the effects of log‐transformed nitrogen addition rate (Nlevel), duration (Nduration), and their interaction on current species richness and community biomass.
**Table S3:** ANOVA table of linear mixed‐effects models (Model 3) testing the effects of log‐transformed nitrogen addition rate (Nlevel), duration (Nduration), rate‐duration interaction, and calendar year on species richness and community biomass under post‐cessation conditions.
**Table S4:** ANOVA table of linear mixed‐effects models (Model 4) testing the effects of log‐transformed nitrogen addition rate (Nlevel), duration (Nduration), rate‐duration interaction, and calendar year on log response ratios (LRR) of species richness and community biomass.

## Data Availability

All data are available through the Environmental Data Initiative data repository at https://doi.org/10.6073/pasta/f2ab91096cb3bb672182ba091cb467c1 (Tilman 2025). All code used in this study is available at the figshare repository with: https://doi.org/10.6084/m9.figshare.32774583.
