# Supplementary material for: Prolonged Nutrient Enrichment Slows the Recovery of Biodiversity and Productivity After Its Cessation
Source: Glob Chang Biol. 2026 Jul 10;32(7):e70981. doi: 10.1111/gcb.70981 (PMC13351789; doi:10.1111/gcb.70981)
Supplement: Supplementary file 1 — Figure S1: Spatial layout of experimental plots (Field C in experiment 002 at Cedar Creek). Figure S2: Temporal dynamics of plant species richness (a–d) and community biomass (e–h) under different nutrient addition rates and cessation treatments. Figure S3: Estimated species richness (a) and community biomass (b) averaged across the three most recent sampling years (2019, 2022, 2023). Figure S4: Temporal trajectories of log response ratios (RR) for species richness (red) and community biomass (blue) under different nutrient addition rates (from top to bottom panels) and cessation treatments (from left to right panels). Values are shown relative to unamended control plots. Figure S5: Species richness (a, b) and community biomass (c, d) across nutrient addition rates after cessation of 10‐year enrichment (a, c) or 32‐year enrichment (b, d). Figure S6: Changes in species richness (a) and community biomass (b) in the first decade following 10 (yellow) or 32 (blue) years of nutrient enrichment, as well as the corresponding changes in unamended control over the same periods (10_Control and 32_Control). Figure S7: Changes in species richness (a) and community biomass (b) in the first decade following 10 (yellow) or 32 (blue) years of nutrient enrichment at different rates. Figure S8: Temporal changes in the relationship between species richness and community biomass under control (a), fertilized (b), and recovery following nutrient cessation (c). Figure S9: Temporal trajectories of species richness and community biomass responses under continuous nutrient enrichment and following nutrient cessation across all nutrient addition rates. Table S1: ANOVA table of linear mixed‐effects models (Model 1) testing the effects of log‐transformed nitrogen addition rate (Nlevel), duration (Nduration), and their interaction on current species richness and community biomass. Table S2: ANOVA table of linear mixed‐effects models (Model 2) testing the effects of log‐transformed nitrogen addi [file GCB-32-e70981-s001.pdf]

Supplementary information for

Prolonged nutrient enrichment slows the recovery of biodiversity and productivity after its cessation

Miao He*[1], Kathryn E. Barry[2], Elizabeth T. Borer[1], Yann Hautier[2], Cristy Portales-Reyes[3], Eric W. Seabloom[1], David Tilman[1,4], Qianna Xu[5], Forest Isbell[1]

[1]Department of Ecology, Evolution, and Behavior, University of Minnesota, St. Paul, MN, USA
[2]Ecology and Biodiversity, Department of Biology, Institute of Science, Utrecht University, Utrecht, 3584 CH, The Netherlands
[3]Department of Biology, Saint Louis University, 3507 Laclede Ave, St. Louis, MO, 63103, USA
[4]Bren School of Environmental Science and Management, University of California, Santa Barbara, Santa Barbara, CA, 93106, USA
[5]Washington State Department of Ecology, 300 Desmond Drive SE, Lacey, WA, 98503, USA

Corresponding author: Miao He
Telephone: (+1) 612-363-2569
Email: miaohe.eco@gmail.com

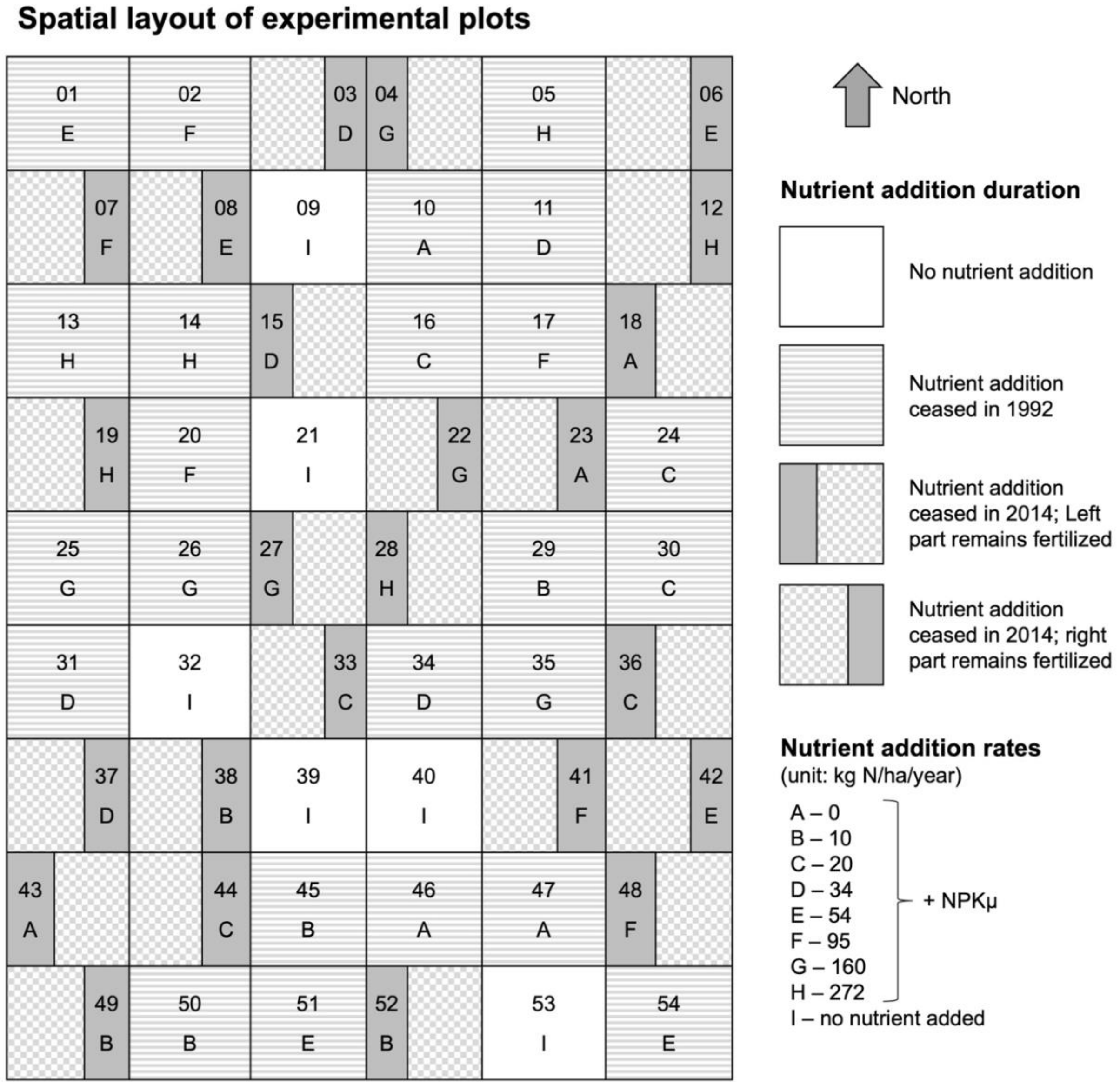

Figure S1 Spatial layout of experimental plots (Field C in experiment 002 at Cedar Creek). Plots are labeled by plot ID and nutrient addition rate (A–I), where I indicates unamended control plots with no nutrient addition (white). All fertilized plots also received P, K, and micronutrients, including treatment A, where no nitrogen was added. Half of the plots ceased nutrient addition in 1992 (striped), the other half of the plots continued to receive nutrient addition until 2014, when those fertilized plots were split into two parts: the 4 m x 2.5 m part (checkerboard) ceased nutrient addition thereafter, and the 4 m x 1.5 m part (dark gray) continues to receive nutrient addition; the position of fertilized split plot (west or east) was randomly assigned by a coin flip.

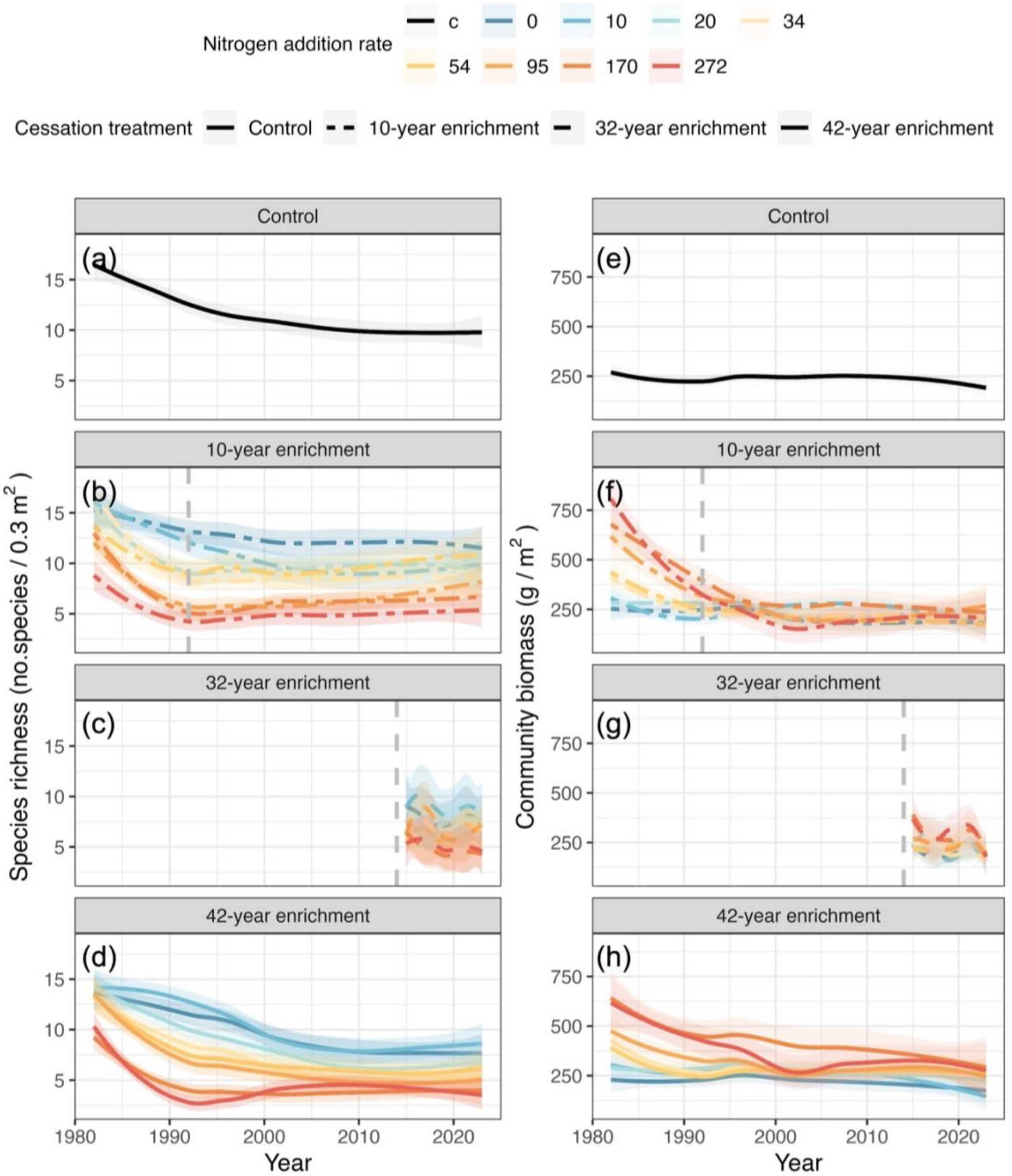

Figure S2 Temporal dynamics of plant species richness (a–d) and community biomass (e–h) under different nutrient addition rates and cessation treatments. Lines show loess-smoothed trends with 95% confidence intervals. Vertical dashed lines indicate the year when fertilization ceased (1992 or 2014, where applicable).

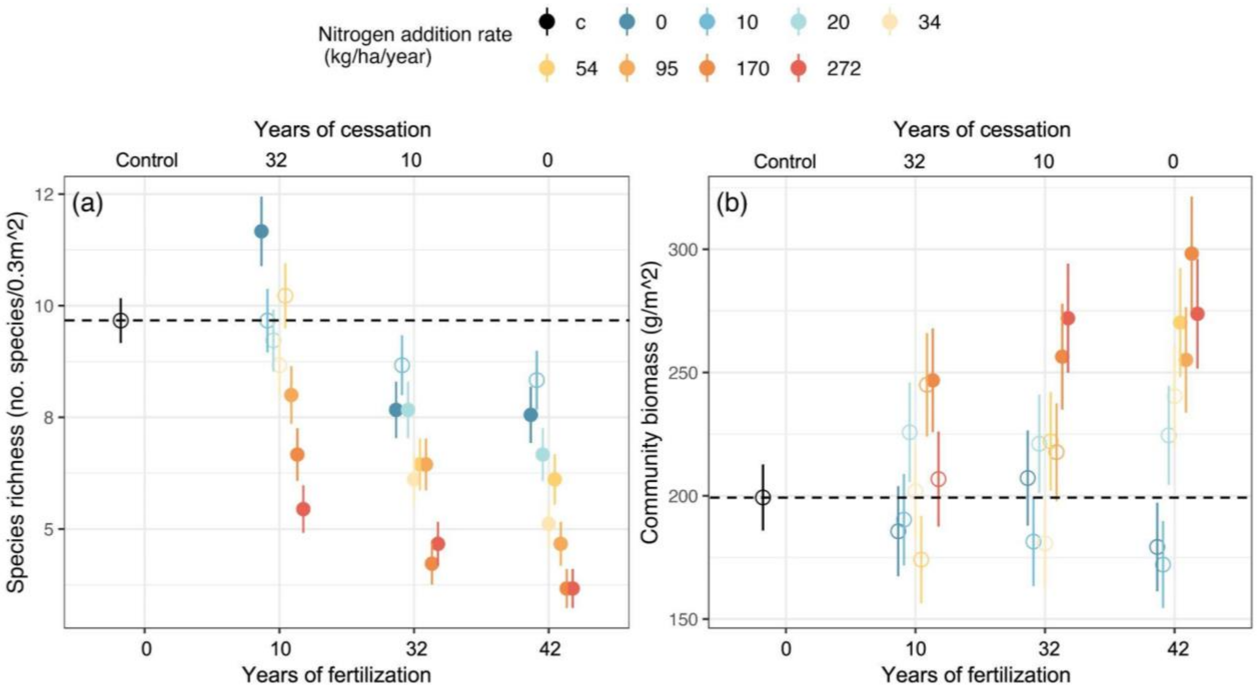

Figure S3 Estimated species richness (a) and community biomass (b) averaged across the three most recent sampling years (2019, 2022, 2023). Points represent model-estimated means, bars represent standard errors under different nutrient rates and cessation treatments. Dashed horizontal lines indicate the unamended control levels (no nutrient was added, distinct from the plots with nitrogen addition rate "0", where no N but other nutrients were added). Solid circles denote treatments that differ significantly from the control, whereas open circles indicate no significant difference, based on Dunnett-adjusted comparisons. Points are jittered for clarity.

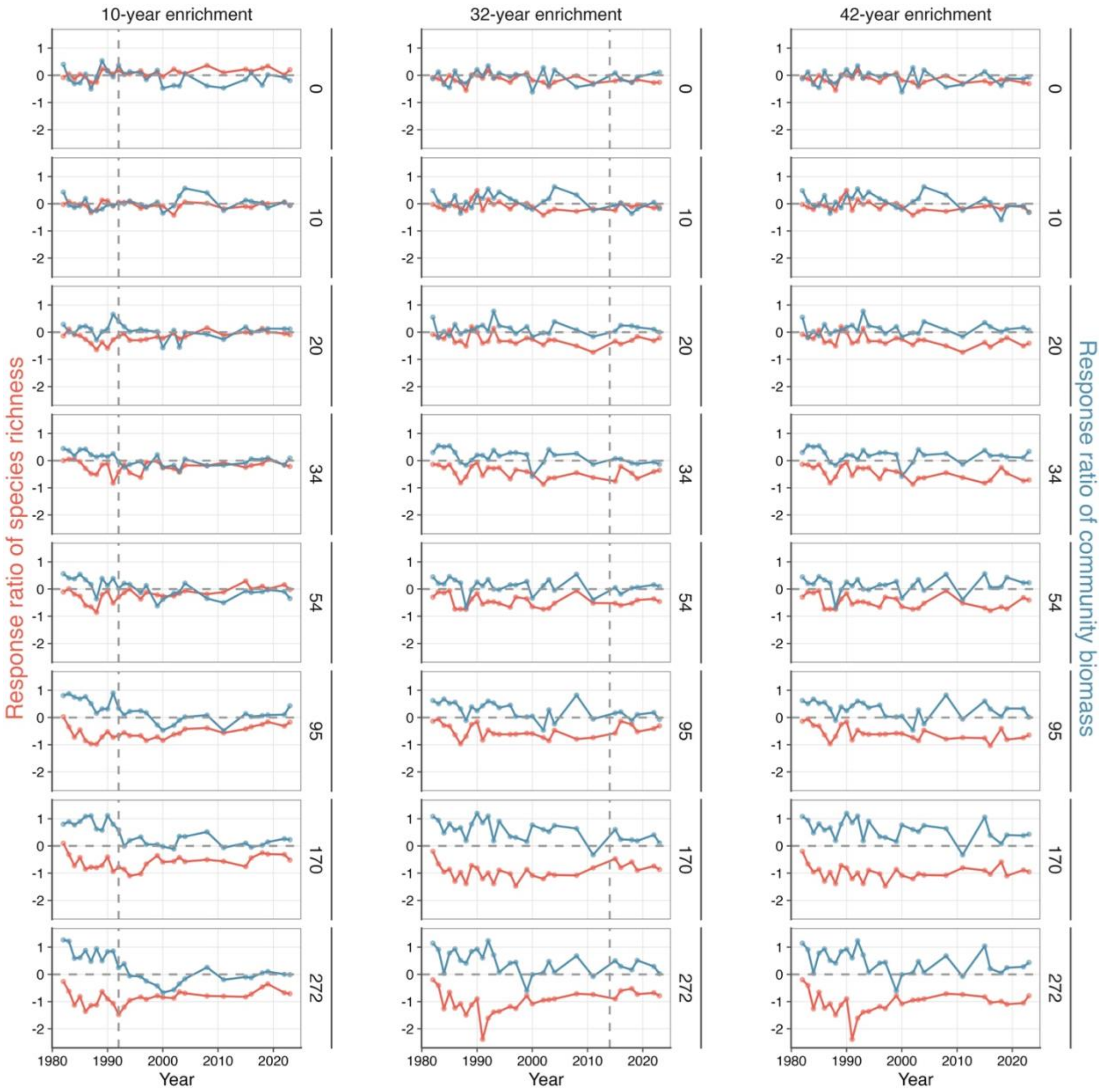

Figure S4 Temporal trajectories of log response ratios (RR) for species richness (red) and community biomass (blue) under different nutrient addition rates (from top to bottom panels) and cessation treatments (from left to right panels). Values are shown relative to unamended control plots.

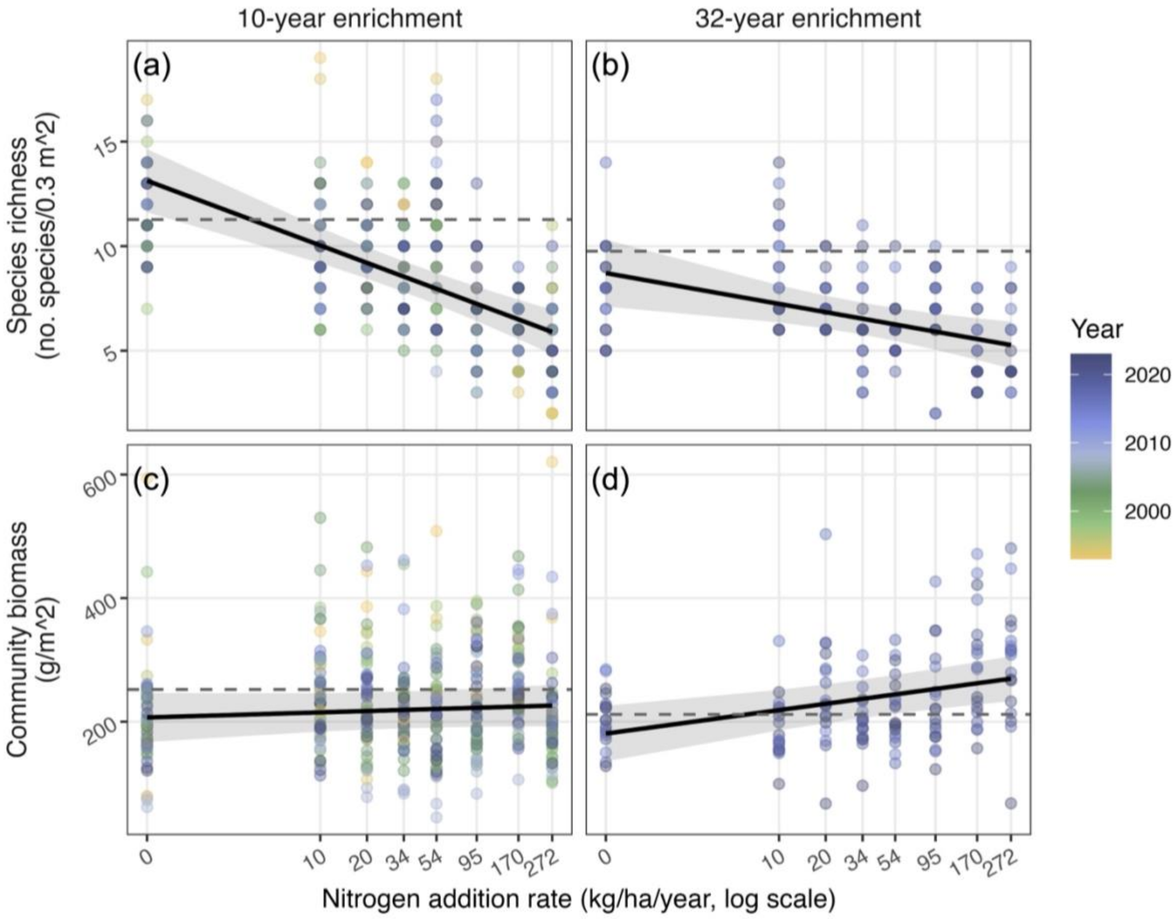

Figure S5 Species richness (a, b) and community biomass (c, d) across nutrient addition rates after cessation of 10-year enrichment (a, c) or 32-year enrichment (b, d). Each point represents an annual observation, with colors indicating the sampling year. Dashed horizontal lines indicate the mean values of unamended control plots over the corresponding post-cessation period for each cessation treatment (average values from 1992 to 2014 as a baseline for 10-year cessation, average values from 2014 to 2023 as a baseline for 32-year cessation).

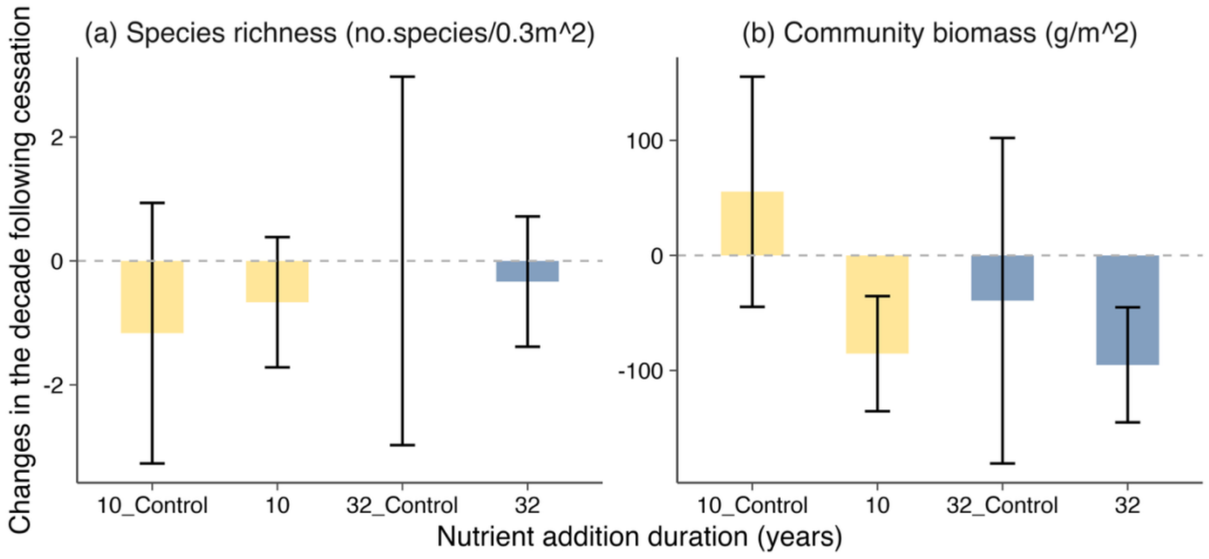

Figure S6 Changes in species richness (a) and community biomass (b) in the first decade following 10 (yellow) or 32 (blue) years of nutrient enrichment, as well as the corresponding changes in unamended control over the same periods (10_Control and 32_Control). Bars (with the standard errors) show differences between model-estimated mean in the later year and those at the cessation year. Dashed horizontal lines indicate cessation-year values; closer to the line indicates less change in the first decade following cessation. Values are centered at zero to represent within-plot changes over the first decade following cessation; baseline values differ among enrichment durations and are shown in Fig. S2.

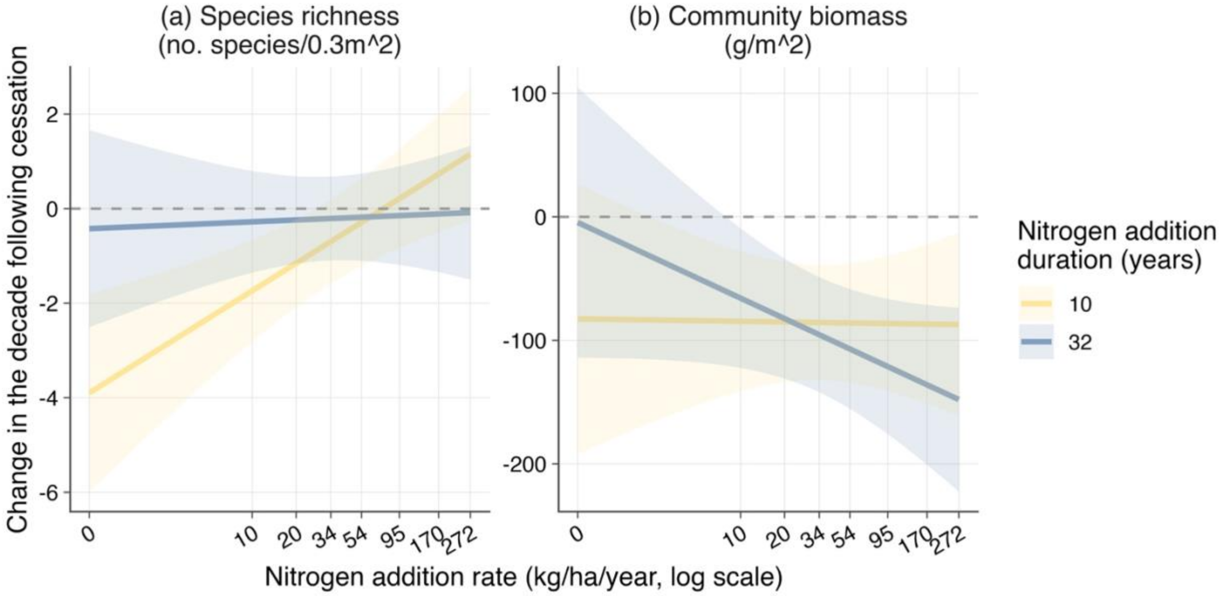

Figure S7 Changes in species richness (a) and community biomass (b) in the first decade following 10 (yellow) or 32 (blue) years of nutrient enrichment at different rates. Lines represent model-predicted changes relative to cessation-year values, and shaded areas indicate 95% confidence intervals. Dashed horizontal lines indicate cessation-year values; closer to the line indicates less change in the first decade following cessation. Values are centered at zero to represent within-plot changes over the first decade following cessation; baseline values differ among enrichment durations and are shown in Fig. S2.

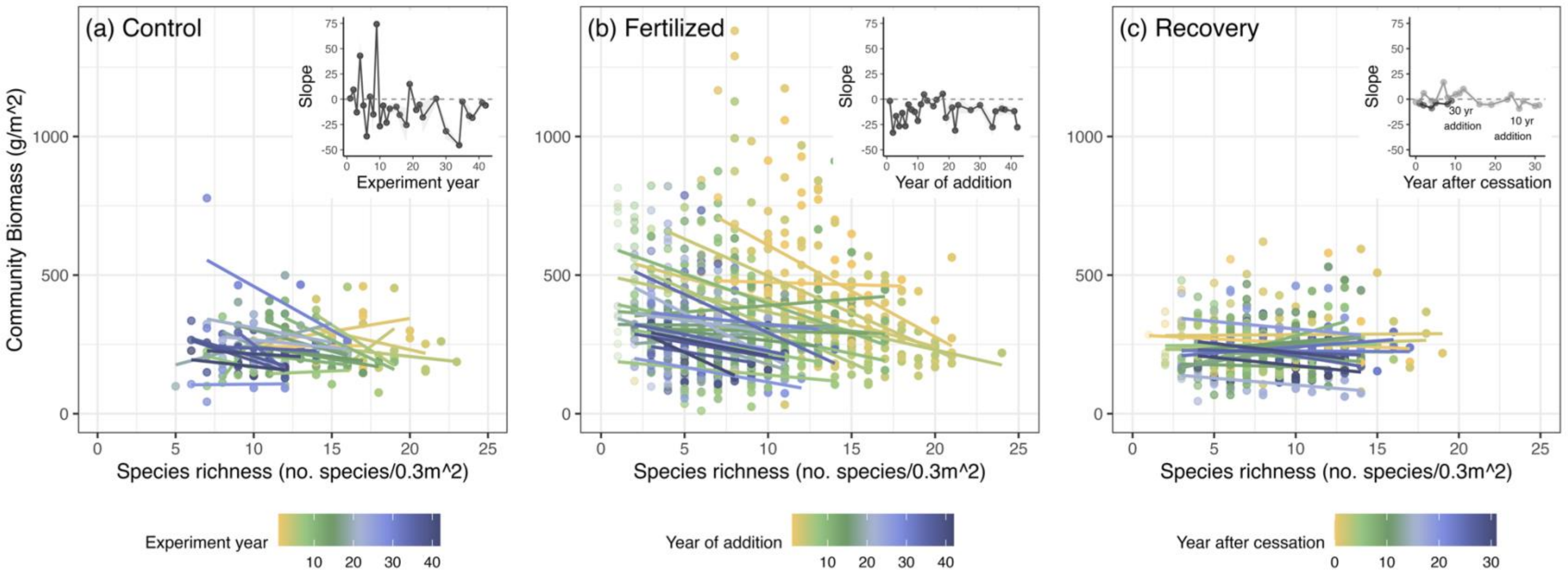

Figure S8 Temporal changes in the relationship between species richness and community biomass under control (a), fertilized(b), and recovery following nutrient cessation (c). Main figures in panels a–c show scatterplots of community biomass against species richness, with points colored by time (experiment year, years of addition, or years following cessation). Lines represent year-specific linear fits. The inserted figures are to quantify temporal changes in the strength of BEF relationships based on the slope estimated separately for each year using linear models lm(biomass~richness), and the shaded areas indicate 95% confidence intervals.

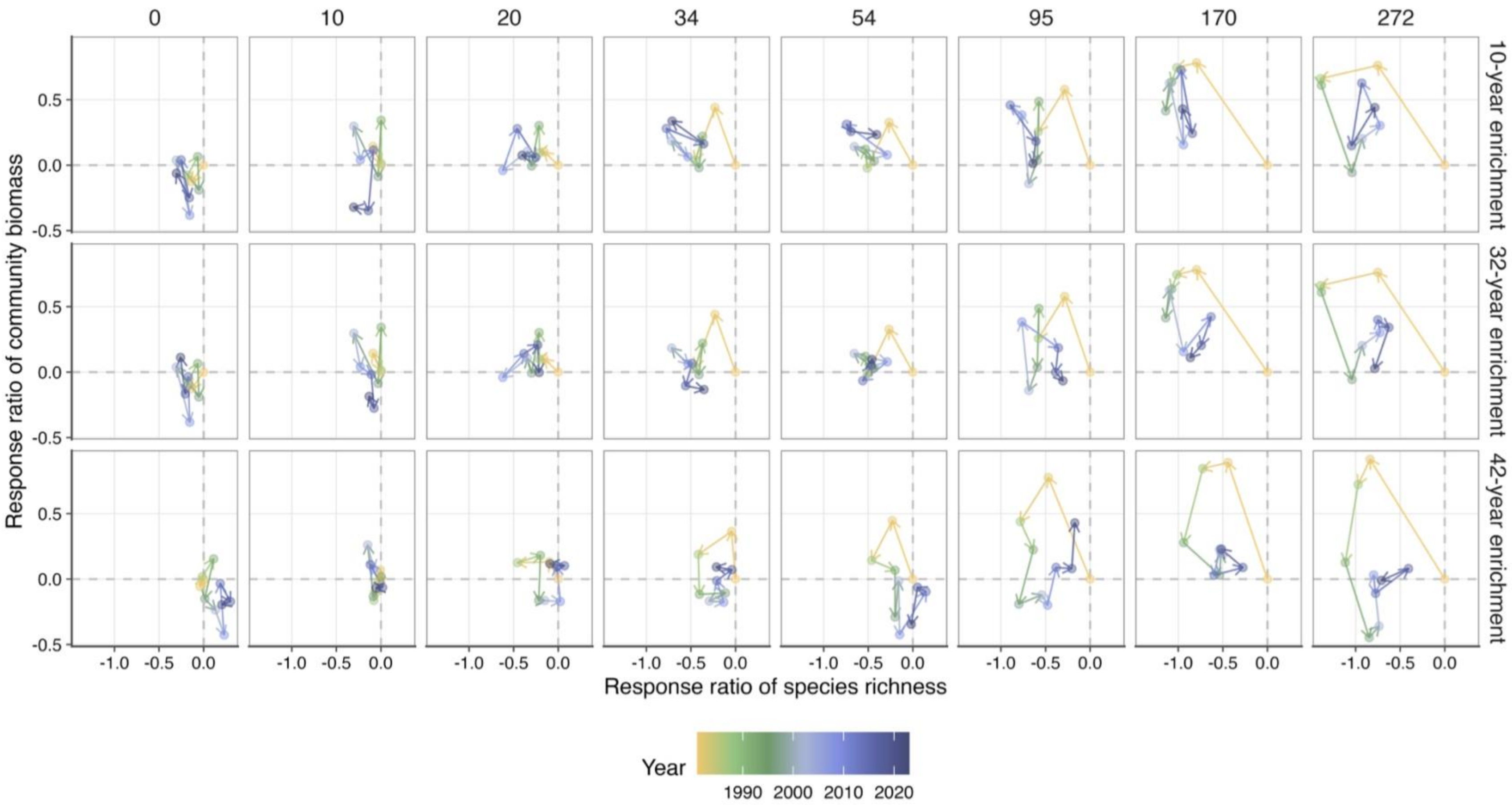

Figure S9 Temporal trajectories of species richness and community biomass responses under continuous nutrient enrichment and following nutrient cessation across all nutrient addition rates. Rows represent nutrient addition duration (10-, 32-, and 42-year enrichment) and columns represent nutrient addition rates (0-272 kg N/ha/year). Responses are shown in a richness–biomass response-ratio space relative to the unamended control (open circles at the origin). Triangles represent the years of cessation, where applicable (d-e). Arrows represent equal time intervals, with color indicating sampling year.

Table S1 ANOVA table of linear mixed-effects models (Model 1) testing the effects of log-transformed nitrogen addition rate (Nlevel), duration (Nduration), and their interaction on current species richness and community biomass. Significance levels are indicated as follows: *** P < 0.001, ** P < 0.01, * P < 0.05, . P < 0.1, ns - not significant.

**Response**	**Predictor**	**Sum Sq**	**Mean Sq**	**NumDF**	**DenDF**	**F value**	**Pr(>F)**	**sig**
Species richness	log(Nlevel + 1)	79.3215	79.3215	1	40.6638	37.2149	<0.001	***
	factor(Nduration)	95.5684	47.7842	2	70.4745	22.4187	<0.001	***
	log(Nlevel + 1): factor(Nduration)	4.5125	2.2562	2	70.4745	1.0585	0.352	ns
Community biomass	log(Nlevel + 1)	57994.8508	57994.8508	1	40.9500	16.6114	<0.001	***
	factor(Nduration)	24363.2108	12181.6054	2	82.8870	3.4892	0.035	*
	log(Nlevel + 1): factor(Nduration)	17853.4852	8926.7426	2	82.8870	2.5569	0.084	.

Table S2 ANOVA table of linear mixed-effects models (Model 2) testing the effects of log-transformed nitrogen addition rate (Nlevel), duration (Nduration), and their interaction on current species richness and community biomass. To assess whether responses deviated from log-linear trends (model 1), both linear and categorical terms for nitrogen addition rate and duration, with rate being log-transformed when used linearly. Significance levels are indicated as follows: *** P < 0.001, ** P < 0.01, * P < 0.05, . P < 0.1, ns - not significant.

Response	Predictor	Sum Sq	Mean Sq	Num df	Den df	F	P-value	sig
Species richness	log(Nlevel + 1)	89.7735	89.7735	1	29.2097	42.0325	<0.001	***
	factor(Nlevel)	25.5526	4.2588	6	29.2097	1.9940	0.099	.
	Nduration	93.1356	93.1356	1	33.9256	43.6067	<0.001	***
	factor(Nduration)	14.0805	4.6935	3	155.3001	2.1975	0.091	.
	log(Nlevel + 1): Nduration	1.5903	1.5903	1	33.9256	0.7446	0.394	ns
	log(Nlevel + 1):factor(Nduration)	11.2165	3.7388	3	155.3001	1.7505	0.159	ns
	factor(Nlevel):YrAdd	9.9425	1.6571	6	33.9256	0.7759	0.594	ns
	factor(Nlevel):factor(Nduration)	33.9616	1.8868	18	155.3001	0.8834	0.599	ns
Community biomass	log(Nlevel + 1)	58529.8056	58529.8056	1	30.4404	17.0541	<0.001	***
	factor(Nlevel)	24857.8338	4142.9723	6	30.4404	1.2072	0.329	ns
	Nduration	13958.1516	13958.1516	1	42.0845	4.0670	0.050	.
	factor(Nduration)	46717.2754	15572.4251	3	155.5221	4.5374	0.004	**
	log(Nlevel + 1): Nduration	9633.8411	9633.8411	1	42.0845	2.8071	0.101	ns
	log(Nlevel + 1):factor(Nduration)	6217.1287	2072.3762	3	155.5221	0.6038	0.613	ns
	factor(Nlevel): Nduration	19532.0650	3255.3442	6	42.0845	0.9485	0.471	ns
	factor(Nlevel):factor(Nduration)	48462.1357	2692.3409	18	155.5221	0.7845	0.716	ns

Table S3 ANOVA table of linear mixed-effects models (Model 3) testing the effects of log-transformed nitrogen addition rate (Nlevel), duration (Nduration), rate-duration interaction, and calendar year on species richness and community biomass under post-cessation conditions. Significance levels are indicated as follows: *** P < 0.001, ** P < 0.01, * P < 0.05, . P < 0.1, ns - not significant.

Response	Predictor	Sum Sq	Mean Sq	Num df	Den df	F	P-value	sig
Species richness	log(Nlevel + 1)	168.4954	168.4954	1	40.6224	55.2154	<0.001	***
	factor(Nduration)	53.0814	53.0814	1	53.5561	17.3946	<0.001	***
	Year	0.1158	0.1158	1	15.4544	0.0380	0.848	ns
	log(Nlevel + 1):factor(Nduration)	18.1479	18.1479	1	45.0136	5.9470	0.019	*
Community biomass	log(Nlevel + 1)	23075.7275	23075.7275	1	29.4770	4.8453	0.036	*
	factor(Nduration)	67.2834	67.2834	1	30.2732	0.0141	0.906	ns
	Year	6870.0381	6870.0381	1	15.0764	1.4425	0.248	ns
	log(Nlevel + 1):factor(Nduration)	20335.9842	20335.9842	1	42.6891	4.2700	0.045	*

Table S4 ANOVA table of linear mixed-effects models (Model 4) testing the effects of log-transformed nitrogen addition rate (Nlevel), duration (Nduration), rate-duration interaction, and calendar year on log response ratios (LRR) of species richness and community biomass. Significance levels are indicated as follows: *** P < 0.001, ** P < 0.01, * P < 0.05, . P < 0.1, ns - not significant.

Response	Predictor	numDF	denDF	F-value	p-value	sig
LRR(species richness)	(Intercept)	1	463	171.9904	<0.001	***
	log(Nlevel + 1)	1	463	1615.6453	<0.001	***
	factor(Nduration)	2	463	60.8773	<0.001	***
	Year	1	26	0.1296	0.722	ns
	log(Nlevel + 1):factor(Nduration)	2	463	8.1295	<0.001	***
LRR(community biomass)	(Intercept)	1	463	25.5127	<0.001	***
	log(Nlevel + 1)	1	463	266.0907	<0.001	***
	factor(Nduration)	2	463	34.7134	<0.001	***
	Year	1	26	6.3009	0.019	*
	log(Nlevel + 1):factor(Nduration)	2	463	18.3579	<0.001	***
